# Numerical modeling and *in situ* small angle X-ray scattering characterization of ultra-small SPION magnetophoresis in a high field and gradient separator†

**DOI:** 10.1039/d3nr05589b

**Published:** 2024-03-06

**Authors:** Xian Wu, Hyeon Choe, Jacob Strayer, Jenifer Gómez-Pastora, Maciej Zborowski, Barbara Wyslouzil, Jeffrey Chalmers

**Affiliations:** a William G. Lowrie Department of Chemical and Biomolecular Engineering, The Ohio State University 151 West Woodruff Avenue Columbus OH 43210 USA chalmers.1@osu.edu +1 (614)292-2727; b Department of Chemical Engineering, Texas Tech University 2500 Broadway Lubbock Texas 79409 USA; c Department of Biomedical Engineering, Cleveland Clinic 9500 Euclid Avenue Cleveland OH 44195 USA; d Department of Chemistry and Biochemistry, The Ohio State University 100 West 18th Avenue Columbus OH 43210 USA

## Abstract

Magnetic nanoparticles (MNPs) have recently gained significant attention in various fields, including chemical and biomedical applications, due to their exceptional properties. However, separating MNPs from solution *via* magnetophoresis is challenging when MNPs are smaller than 50 nm as Brownian forces become on the order of the magnetic forces. In this study, we successfully separated small MNPs (5–30 nm) by utilizing high magnetic fields and gradients generated by economical permanent magnets. *In situ* small angle X-ray scattering (SAXS) was used to investigate the time-dependent concentration changes in the ferrofluid, and the results validated that only the 30 nm particles experienced particle aggregation or agglomeration, indicating that dipole–dipole interactions did not play a discernable role in the separation process for particles smaller than ∼15 nm. However, numerical simulations have provided further validation that in the absence of particle–particle interactions, even MNPs with diameters less than 15 nm exhibited magnetophoresis that effectively counteracted the effects of Brownian motion.

## Abstract 2

Ultra-small SPIONs were successfully separated using high magnetic fields and gradients. *In* s*itu* SAXS and numerical simulation were used to investigate the magnetophoresis process.

## Graphical abstract

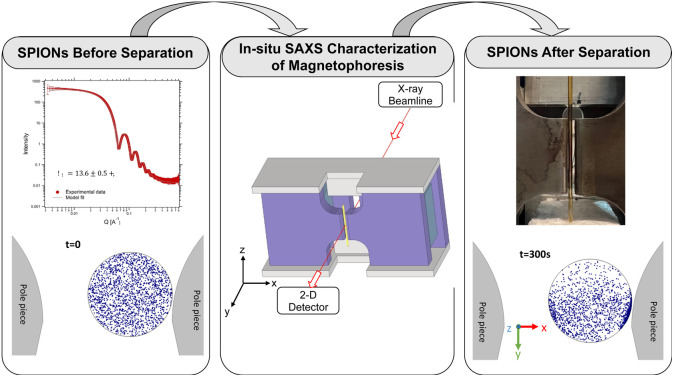

## 1. Introduction

Over the past two decades, magnetic nanoparticles (MNPs) have gained great attention due to their unique chemical and physical properties. By definition, MNPs represent a wide class of materials including particulates that have one dimension less than 100 nm.^1,2^ These nanoscale materials often manifest properties that differ from those of the bulk state due to the significance of surface and quantum confinement effects, among others.^3^ These factors can affect the chemical reactivity of these materials as well as their mechanical, optical, electrical, and magnetic properties.^4–6^ Advances in nanotechnology and particle synthesis allow for the fabrication of MNPs with controllable size, shape, stability, composition, and magnetic response.^4,6–8^ MNPs are generally composed of magnetic elements, such as iron, cobalt, nickel, or their oxides like magnetite (Fe_3_O_4_), maghemite (γ-Fe_2_O_3_), nickel ferrite (NiFe_2_O_4_), and cobalt ferrite (CoFe_2_O_4_),^9–11^ They are usually coated with layers of organic or inorganic materials to stabilize them against oxidation, corrosion, and spontaneous aggregation, as well as to provide a functionalizable surface. These materials exhibit a high surface-area-to-volume ratio, biocompatibility, and most importantly, they can be manipulated by magnetic fields.^6,12,13^ When the dimensions of MNPs fall below specific critical thresholds, which vary depending on material parameters,^14^ magnetic materials transition into a superparamagnetic state. Consequently, in the absence of a magnetic field, nanomaterials in this state lose their magnetic properties, thereby removing magnetism as a source of mutual attraction. This phenomenon offers the advantage of minimizing the risk of particle aggregation while still displaying a robust response to an external magnetic field.^15–17^ With these outstanding properties, MNPs have been broadly used within the medical field in targeted drug delivery systems as magnetic resonance imaging (MRI) contrast agents and for the biomolecular separations of proteins, nucleic acids, and cells.^18–20^

In magnetic field-assisted bio-separations, target molecules bind to MNPs *via* affinity ligands, creating a particle-biomolecule complex that can then be separated using a magnetophoretic device.^21^ However, even the most advanced magnetic separation systems present challenges for nanoparticle separation. Firstly, due to the linear relationship between the magnetic force and the particle volume, high magnetic field gradients are required to separate MNPs smaller than ∼30 nm from solution. Therefore, high gradient magnetic separation systems (HGMS) must be employed. Conventional HGMS filters usually operate with electromagnets, making the operation expensive due to their high energy, and typically cooling requirements.^22,23^ Moreover, the use of electrical currents to generate the magnetic field also causes Joule heating, which may degrade the sample. HGMS filters also utilize a matrix (ferromagnetic filaments and spheres) to generate high magnetic gradients, and these matrices can both trap non-magnetic solids and damage the biomaterial of interest. For example, if cells are being separated, they can be contaminated or destroyed by direct exposure to the matrix or ions leached from the matrix.^24,25^ Uncertainty regarding the magnetic and hydrodynamic conditions inside the filter results in a limited understanding of the process and hinders rational design and optimization.^4,26,27^ In contrast, matrix-free devices based on permanent magnets are not optimized and generate only relatively low fields (1 T) and gradients (smaller than 100 T m^−1^), the so-called low gradient magnetic separation (LGMS).^28–30^ Consequently, MNP recovery can require several hours/days of operation to carry out the separation.^31–33^ Finally, MNP magnetophoresis is a complex process and the physics behind it is not completely understood.^34^ Many forces and interactions are involved,^35,36^ and these are often difficult to describe analytically.

Small-angle X-ray scattering (SAXS) is a highly effective technique that can determine the size distribution parameters, morphology, and surface structure of nanoparticles.^37^ Moreover, SAXS offers valuable insights into interparticle interactions by exploring interparticle correlations within aggregates and the formation process of any superstructures.^38^ The advent of advanced synchrotron X-ray sources featuring high energy and flux, along with state-of-the-art detectors, has facilitated the widespread use of SAXS to investigate a diverse range of nanoparticulate systems.^39^ In general, photons scattered by the sample within the range of 0.1 to 5 degrees are collected using a two-dimensional detector. These data are then binned and averaged to produce the one-dimensional scattering spectra *I*(*Q*), where 
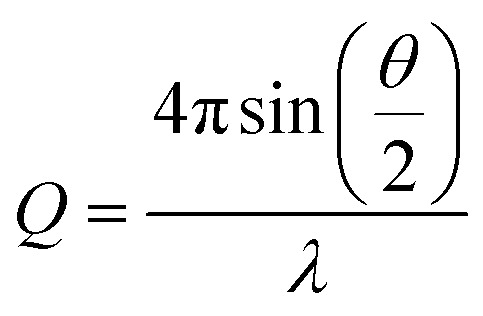
 is a function of both the scattering angle, *θ*, and the wavelength, *λ*, of the incident X-rays. Furthermore, SAXS can be used to conduct dynamic experiments, such as investigating the *in situ* development of nanostructures under various conditions, including in the presence of magnetic fields. Here, we use SAXS to characterize nanoparticle suspensions as a function of time and position within a strong magnetic field and magnetic field gradients generated by a permanent magnet-based device. Our goal is to quantify the kinetics of the separation process better and to detect possible particle–particle interactions that result from the strong magnetic fields and magnetic field gradients.

The current work complements our recent publications, where we used time resolved photography to experimentally demonstrate the manipulation or separation of 5–30 nm MNPs using permanent magnets.^40,41^ There we suggested that the separation occurred by a combination of magnetic forces, magnetic dipole–dipole interactions, and gravitational forces. More precisely, it was hypothesized that the particles underwent agglomeration through dipole–dipole interactions, resulting in the formation of aggregates large enough to experience significant gravitational forces, causing them to sediment in our quadrupole magnets. In that work, however, we could not quantify the role (if any) of these effects in the separation process or determine if (pre-) aggregation of the sample played a role. The goal of this work is to start to unravel the mechanisms at play when ultra-small (as small as 5 nm) MNPs are separated using high magnetic fields and gradients. We employ a novel, permanent magnet-based system that integrates multiple permanent magnets in specific arrangements to yield fields and gradients with strengths higher than those provided by conventional permanent magnet devices. Complementary modeling, simulation, and experimental validation of the magnetic fields generated in the system are also conducted. Commercial superparamagnetic iron oxide nanoparticles (SPIONs) suspended in toluene are the target MNPs to be separated and time-resolved SAXS experiments are used to follow local changes in MNP concentrations in the solution, and to look for any evidence of interactions between the MNPs. The initial findings indicate that nanoparticles within the 5–15 nm range do not undergo particle–particle interactions that result in segregation or aggregation. These findings may contradict our hypotheses based on previous photographic measurements. However, the information provided in this study can guide the optimization of MNP separation with permanent magnets and may eventually serve to decrease particle dosage and size requirements in general MNP applications.

## 2. Theory

When SPIONs are exposed to a magnetic field, the motion of the particles is influenced by several forces, including the magnetophoretic force (*F*_m_), the gravitational force (*F*_g_), the drag force (*F*_d_), and the Brownian force (*F*_b_). The equations that describe the motion of the particles are as follows:1
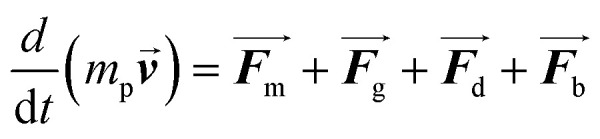
2
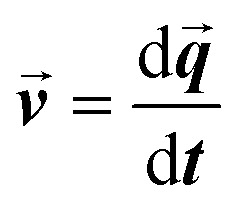
where *m*_p_ represents the mass of the particle, 
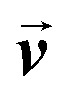
 is the particle velocity, and 
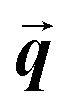
 is the position of the particle.

When nanoparticles are subjected to an inhomogeneous magnetic field, the *F*_m_ acting on the particles is given as follows:^42^3
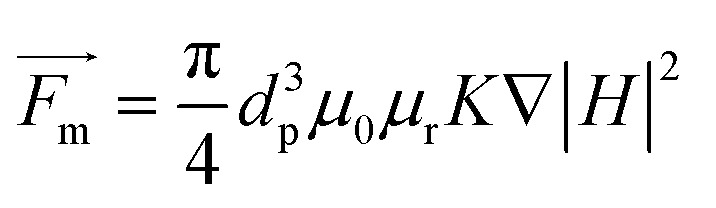
where4
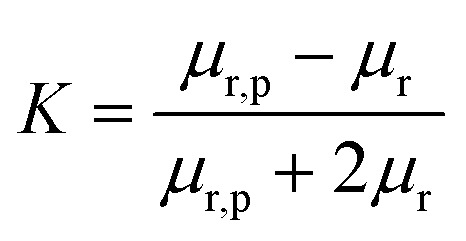
*d*_p_ is the particle diameter, *μ*_0_ is the magnetic permeability of vacuum, *μ*_r_ is the fluid relative permeability, *μ*_r,p_ is the particle relative permeability, and *H* is the magnetic field intensity.

The net *F*_g_ acting on a spherical nanoparticle is given by:5
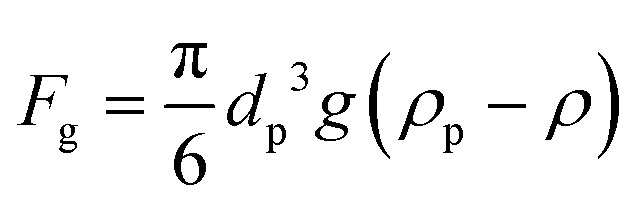
where *g* is the acceleration due to gravity (9.8 m s^−2^), *ρ*_p_ is the density of the particle and *ρ* is the density of the surrounding fluid.

As the particle moves in a medium, it experiences an opposing viscous drag force, which can be estimated as follows:6
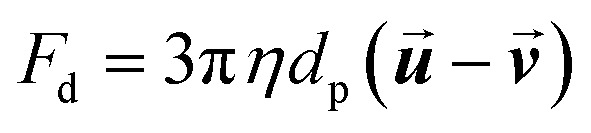
where *η* is the fluid viscosity, and 
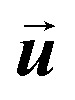
 is the fluid velocity.

Moreover, for a particle undergoing classical Brownian motion, *F*_b_ describes the force applied by the surrounding solvent molecules onto the particle that can be expressed as:^43^7
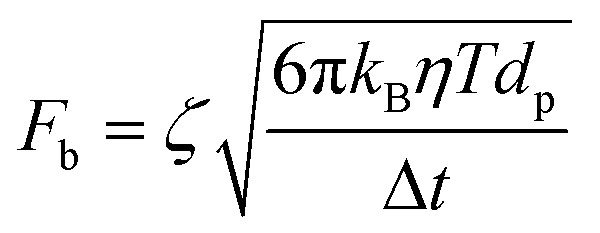
where *ζ* is a normally distributed random number with a mean of zero and unit standard deviation, *k*_B_ is the Boltzmann constant (1.38 × 10^−23^ J K^−1^), *T* is the temperature, and Δ*t* is the time step considered (10^−8^ s in this work).

In the presence of a magnetic field, particles suspended in a fluid tend to be drawn towards regions of higher magnetic field intensity, in the direction of the magnetic field gradient. However, the random thermal motion of the particles counteracts this magnetic force and enables their diffusion throughout the fluid. The stability of these particle suspensions against separation in a magnetic field can be predicted by comparing the relative strength of the magnetic energy and thermal energy. A higher ratio between these energies suggests that particles will overcome thermal motion and can migrate or maintain their position in the magnetic field. The ratio between the magnetic energy and the thermal energy (*ε*) is expressed as:^36^8
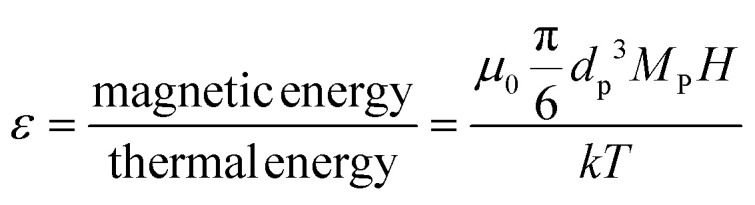
where *M*_P_ is the saturation magnetization of the particle.

The magnetic behavior of the SPIONs can also be influenced by magnetic dipole–dipole interactions that enhance the formation of agglomerates. Thermal agitation can disrupt these agglomerates, and the ratio between the dipole–dipole contact energy and thermal energy, *Ψ*, is expressed as:9
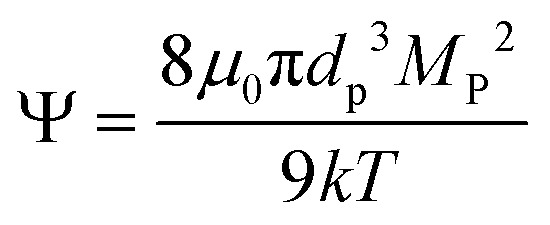


To investigate the magnetic separation of SPIONs of varying sizes under the influence of applied magnetic fields, this study examines the forces acting on the particles, along with various energies. The subsequent sections present a detailed analysis of these factors, offering a possible explanation for the experimental observations and the underlying separation mechanisms.

## 3. Materials and methods

### 3.1 Materials

The nanoparticles used here are SPIONs originally suspended in chloroform. They were obtained from Ocean Nanotech (San Diego, CA, USA) and correspond to particles with nominal sizes of 5 nm (SOR05-02), 15 nm (SOR15-02) and 30 nm (SOR30-02). The SPIONs are coated with oleic acid to prevent particle aggregation and to enable the particles to remain uniformly dispersed in the solvent. The stock solutions all have a concentration of 25 g L^−1^.

To minimize the impact that chloroform would have on the background in the X-ray scattering experiments, solvent exchange was performed on the SPION suspensions to replace the chloroform with toluene. The solvent exchange process was as follows: 2 mL of acetone (Sigma-Aldrich, 99.8% purity) was added to 1 mL of the stock SPION solution to precipitate the particles. After centrifugation, the precipitate was resuspended in 1 mL of toluene (Sigma-Aldrich, 99.9% purity) to achieve the same concentration of 25 g L^−1^ for further dilutions.

For the magnetic separation experiments and SAXS measurements, SPIONs were diluted in toluene to a concentration of 12.5 g L^−1^. To ensure good background subtraction, X-ray scattering was first measured at selected locations on the quartz capillary tubes (1.48 mm i.d., 1.5 mm o.d., Charles Supper, catalog number Quartz 15-QZ) containing only toluene. The toluene was then removed from the capillary tubes, and the SPION suspensions were introduced up to a height *h* = 4.5 cm. The capillaries were sealed with epoxy to prevent evaporation of the toluene. SAXS measurements were then made at the same locations as the background measurements.

### 3.2. Magnet system

**Fig. 1:**
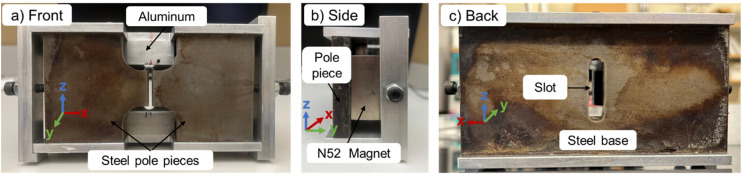
The magnetic separation system. (a) Front view shows the steel pole pieces that conduct the magnetic flux across the air gap and the aluminum frame used to assemble the pieces. (b) Side view shows how the magnets are located between the steel pieces. (c) Back view shows the slot that provides X-ray access to the sample.

The design of our magnetic separation system using permanent magnets has been presented before.^44,45^ Briefly, the design consists of two rectangular-shaped permanent magnets (50.8 mm wide × 50.8 mm long × 25.4 mm deep, NdFeB, Grade N52, K&J Magnetics) held between a steel base and two steel pole pieces (low carbon steel, grade 1018). The two steel pole pieces are used to conduct the magnetic flux. In theory, this design allows the magnetic flux to travel from the block magnets into one of the pole pieces, move across the air gap, enter the second pole piece, and finally return to the block magnets. For the *in situ* SAXS measurements, a slot (38.1 mm × 9.525 mm) was cut into the back steel piece to allow the X-ray beam to pass through the sample (see Fig. 1). A CAD drawing of this device is available in the ESI as Fig. S1.†

### 3.3 Finite-element method simulations of the magnetic field and particle path

The finite-element method (FEM) simulation software, COMSOL Multiphysics 6.0,^46^ was used to numerically model the magnetic field and determine the particle trajectories inside our system. Specifically, the “magnetic fields, no currents” (mfnc) physics module within the AC/DC module was selected to model the magnetic flux density (*B*) and magnetic field density (*H*). COMSOL simulations were conducted at the Ohio Supercomputer Center using 2 nodes.^47^ Laboratory measurements of the magnetic field were conducted using a model 455 DSP gaussmeter (Lakeshore Cryotronics) to compare the experimentally measured fields and the simulation results.

**Fig. 2:**
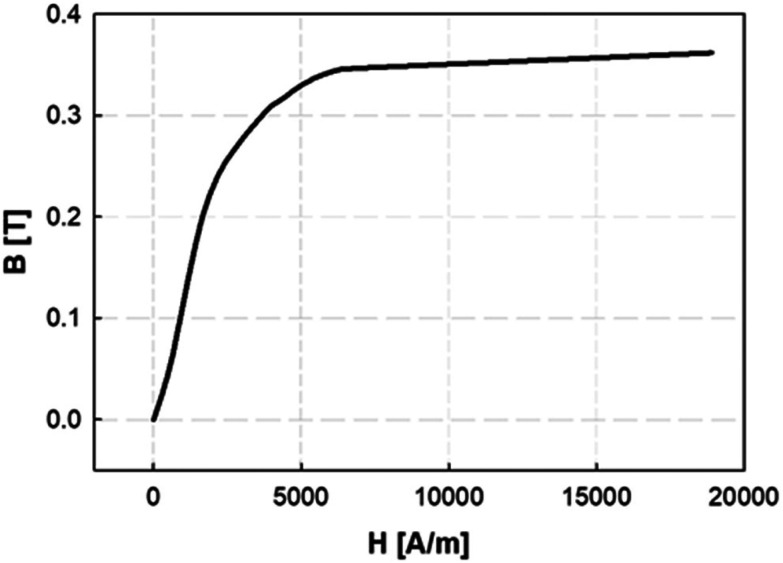
*B*–*H* curve for low-carbon steel magnetite used in the numerical simulations.

To study the SPIONs' behavior inside the magnet system, the “particle tracing for fluid flow” package in COMSOL was selected. It was not possible to model the SPIONs used in the experiments with complete accuracy because a measured magnetization curve was not available. Instead, the simulations assumed that the SPIONs are made of low-carbon steel magnetite. The bulk density of this material is 5000 kg m^−3^, and the magnetic *B*–*H* curve provided by the software is depicted in Fig. 2. Magnetophoretic, gravitational, Brownian, and drag forces were all included in the model. The magnetophoretic force was determined based on the particles' magnetic properties and the magnetic field's magnitude and direction (eqn (3)). The gravitational force (eqn (5)) was determined based on the density of the particles relative to that of the fluid (*ρ* = 867 kg m^3^). The Brownian force was determined based on the size of the particles and the temperature of the system (eqn (7)). The drag force (eqn (6)) was determined based on the fluid velocity and the dynamic viscosity of toluene (*η* = 0.548 mPa s). Additionally, we employed a bounce wall condition, meaning that particles rebounded if they collided with a wall. A time-dependent solver was utilized, with an initial time step of 10^−6^ s, and the positions and velocities of the particles were updated at each time step according to the forces acting on them. The particle trajectories were tracked with time to evaluate particle behavior within the magnetic field. A final time of 300 s was simulated. The separator was simulated in 3-D with over 7 million mesh cells.

### 3.4 SAXS measurements of the particle suspensions in the system

SAXS measurements were carried out at the 12-ID-B beamline of the Advanced Photon Source (APS), Argonne National Laboratory (Lemont, IL). The beam energy was 13.3 keV, the beam dimensions were roughly 0.8 mm (horizontal) × 0.2 mm (vertical), and each measurement exposed the sample to a single X-ray shot for 0.1 s. The scattered photons were collected using a Pilatus 2 M two-dimensional detector. One-dimensional scattering profiles were obtained by integrating the two-dimensional scattering patterns at constant *Q*.^51^

**Fig. 3:**
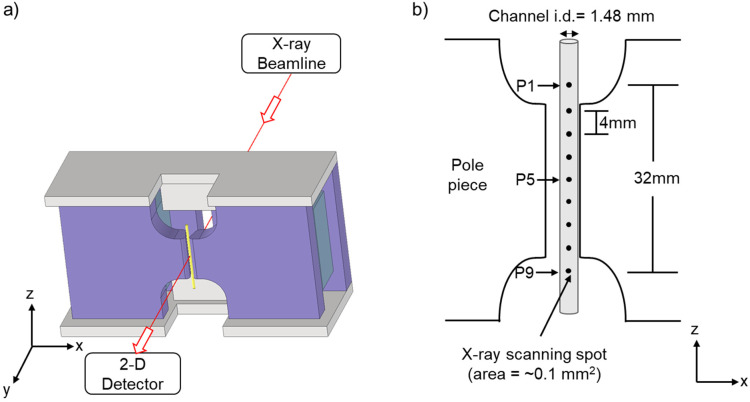
Schematic representation of the experimental setup for the *in situ* SAXS measurements that shows (a) the magnetic separator and the 1.48 mm ID capillary containing the nanoparticle solution in the X-ray beam (area = ∼0.1 mm^2^), and (b) the nine designated positions along the tube length at which SAXS measurements were made.

For the *in situ* X-ray scattering measurements, the tube containing the SPION suspension was placed within the magnetic separator between the two pole pieces, as illustrated in Fig. 3. SAXS measurements were made at 9 evenly spaced positions along the tube length corresponding to a total length of 32 mm. As shown in Fig. 3(b), 7 positions were located within the narrow gap between the pole pieces, and 2 positions were outside, at the top and bottom. The first measurements were made as soon as possible after the tube was placed in the magnet (≲3 minutes), and subsequent measurements were taken at time intervals that varied with the particle size under investigation.

The data were corrected for background scattering arising from the glass capillary and toluene. The results were analyzed using Igor Pro (WaveMetrics Inc.) and the Irena software package^48^ to obtain particle size distribution parameters, relative volume fraction, as well as any structural information that may arise from strong particle–particle interactions during separation. To establish the uncertainty in the volume fraction derived from SAXS, a separate set of experiments measured samples that had not been exposed to a magnetic field. For a given sample, the volume fractions measured at the 9 positions within the capillary tube varied by less than 6% from the average volume fraction.

## 4. Results

### 4.1 Magnetic separator field map

**Fig. 4:**
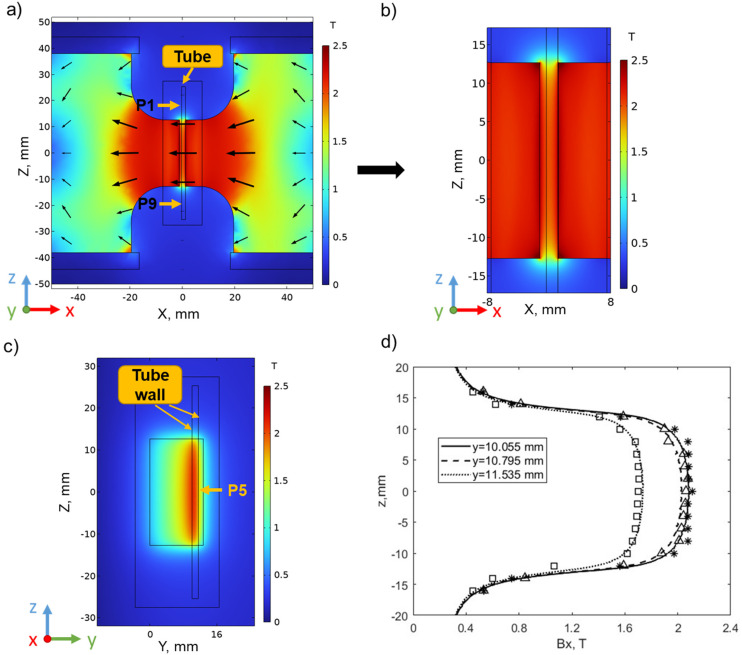
Magnetic flux density color maps determined numerically for (a) the *x*–*z* plane on the front face of the pole plates (*y* = 10.795 mm) and (b) an enlarged view of the color map for the region in between the pole pieces. The origin of the coordinate system is along the centerline of the gap between the pole plates and in line with the back face of the 12.7 mm thick pole plates. (c) Magnetic flux density color maps for the *y*–*z* plane at *x* = 0.4 mm. (d) The theoretical predictions of *B*_*x*_ (lines) agree well with the experimental measurements (symbols) along the *z*-axis for *x* = 0.4 mm and three different *y* locations.

The magnetic field characteristics are depicted and illustrated in Fig. 4. Fig. 4(a) illustrates the predicted distribution of magnetic field flux density magnitude (*B*) within the pole pieces in the *x*–*z* plane obtained from the simulations, where *y* = 0 is located on the back face of the pole plates. The field intensity reaches a maximum of 2 T within and between the pole pieces. The intensity gradually diminishes above and below the pole pieces, resulting in fields on the order of 0.5 T. Fig. 4(a) also shows the direction of the magnetic flux lines between the pole pieces, indicating that particles positioned within the magnetic separator experience magnetic forces in the *x*-direction. Fig. 4(b) displays the magnetic field flux density distribution between the pole pieces in the *y*–*z* plane and demonstrates that the design of the pole pieces also leads to a field gradient in the *y*-direction.

To quantitatively assess the field intensity predictions, Fig. 4(c) compares the calculated and measured magnetic field distributions in the *x*-direction, *B*_*x*_, as a function of the vertical position *z* for three different values of *y*. The simulation data are represented by lines and the experimental measurements made using the gaussmeter are represented by the symbols. The chosen simulation positions correspond to the front (*y* = 11.535 mm) and the back of the tube wall (*y* = 10.055 mm), and the middle of the tube (*y* = 10.795 mm) where the SPION suspensions are contained. Measurements using the gaussmeter were made to match these positions as closely as possible. All the measurements and calculations were made at *x* = 0.4 mm, which is the center of the tube when placed against the side of the pole of the magnet, as illustrated in Fig. 3(b). The good agreement between the experimental data and the predictions confirms the accuracy of the numerical model.

### 4.2. SAXS measurements

**Fig. 5:**
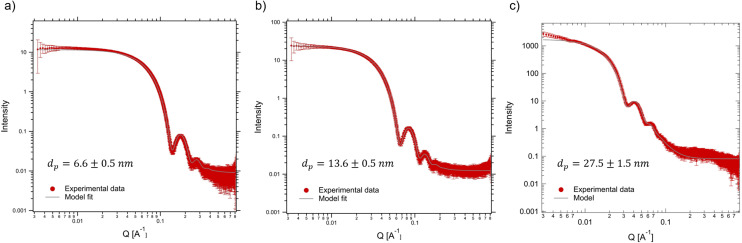
SAXS intensities obtained for our particle suspensions in the absence of a magnetic field and the corresponding model fits. (a) 6.6 nm, (b) 13.6 nm and (c) 27.5 nm SPION suspensions.

To confirm the homogeneity of the nanoparticle suspensions with respect to size, stability (lack of pre-aggregation), and uniform distribution in the suspension prior to magnetic field exposure, we first performed SAXS measurements on diluted suspensions of SPIONs (12.5 g L^−1^) in the absence of a magnetic field. The resultant spectra, measured at position 1, are shown as a function of the scattering vector in Fig. 5. The raw data correspond to the red points (with error bars), while the grey solid lines are the best fits assuming a polydispersed collection of spheres that follow a Gaussian distribution. The fits yield particle sizes consistent with the manufacturer's specifications and confirm that the particles are quite monodispersed and essentially free of aggregation. Only the largest particle size shows a slight increase in intensity at low *q* that may arise from particle pre-aggregation or due to incomplete background subtraction at low *q*. In particular, particles labeled as “5 nm” have a measured diameter of 6.6 ± 0.5 nm, those labeled as “15 nm” have a measured diameter of 13.6 ± 0.5 nm, and the “30 nm” particles have a diameter of 27.5 ± 1.5 nm. The particle sizes found by fitting the SAXS data were used as the input for our particle tracking simulations discussed below.

To investigate SPION separation under high magnetic fields, each SPION solution was exposed to the magnetic field for the duration specified in Table 1 (Total time). The 0.1 s X-ray scattering measurements were made intermittently at the time intervals summarized in Table 1. For example, in Exp #1, the solution remained in the magnetic separator for 10.5 hours. SAXS measurements were made as soon as possible after placing the capillary tube in the magnetic field (*t* = 0 minutes) and then after 30-minute intervals until the end of the experiment. Fig. S2 in the ESI† shows the SAXS spectra measured at position 1 for the particle suspensions at the end of each experiment, and where possible, the corresponding model fits.

**Table 1:** Summary of the SAXS measurement intervals for magnetic separation experiments

Exp #	Particle size (nm)	Total time in the magnetic field	Time intervals for SAXS measurements
1	6.6	10.5 h	Every 30 min
2	13.6	100 min	Every 5 min
3	27.5	30 min	Every 5 min

**Fig. 6:**
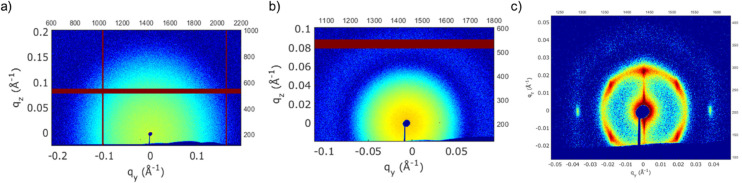
Typical 2-dimensional SAXS patterns for SPION solutions exposed to external magnetic fields at position 5 for the maximum experimental time noted in Table 1. This position corresponds to the highest B-field. Particle sizes are (a) 6.6 nm, (b) 13.6 nm and (c) 27.5 nm. (Dark red-brown lines are masked portions of the detector).

Fig. 6 presents typical 2-dimensional SAXS patterns observed for the three particle sizes after separation at the total exposure times listed in Table 1. The dark red/brown lines in the SAXS patterns are masked points. For the two smaller particle sizes, 6.6 and 13.6 nm, the SAXS patterns appeared to be isotropic, consistent with the absence of any locally ordered structures in the suspension. Furthermore, all the 1-dimensional spectra resembled those shown in Fig. 5 (see also Fig. S3 and S4†). In other words, there was no evidence of particle–particle aggregation or agglomeration despite the relatively long time spent under the influence of the magnetic field. In contrast, the 2-dimensional scattering pattern observed for the 27.5 nm particles (Fig. 6c) is consistent with the development of a Bravais type lattice,^49^ indicating that these particles aligned themselves due to the dipole–dipole attraction induced by the external magnetic field. Furthermore, within a remarkably short period of 3 minutes, the 27.5 nm particles had already rapidly separated and migrated towards the magnet pole pieces, forming a concentrated layer near the channel wall. With time, the solution in the center of the tube became progressively clearer, with visible deposition on the sidewalls. There was no evidence of sedimented aggregates at the bottom of the tube. Furthermore, as shown in Fig. S2,† it was difficult to adequately fit the SAXS data for the 27.5 nm SPIONs after the separation process, primarily due to the emergence of a superstructure. Consequently, these data were excluded from further analysis.

**Fig. 7:**
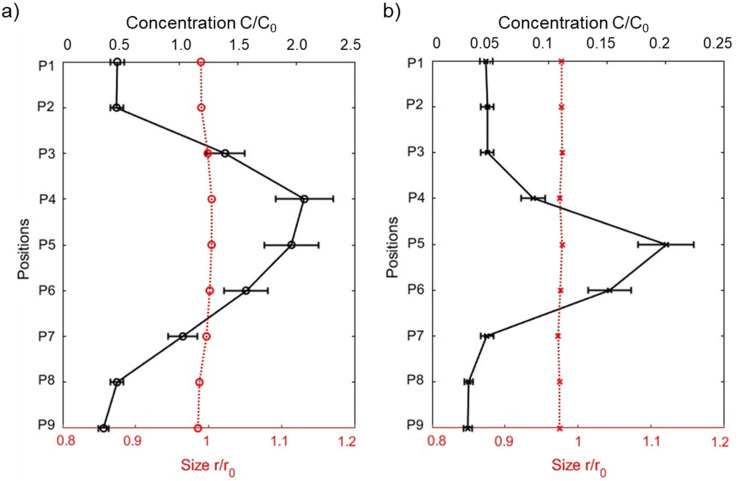
Ratios between the suspension concentration of SPIONs (solid line, top axis) and the size of SPIONs (dash line, bottom axis) after separation at various scanning positions, relative to the initial concentration or size (*C*_0_ or *r*_0_) at *t* = 0. The results are presented for SPION sizes of (a) 6.6 nm and (b) 13.6 nm. Representative error bars on *C*/*C*_0_ are based on an estimated uncertainty of 6% in determining the volume fraction.

From the fitting of the 1-D spectra for the two smaller particle sizes, there was essentially no change in the particle size as a function of time or position in the tube, even though the concentrations did change, as evidenced by the shifting in the intensities of the spectra (see Fig. S3 and S4†). Fig. 7 shows that the ratio of the SPION size at the longest exposure time to that prior to exposure (*t* = 0) changed by less than 3%. In contrast, the ratio of SPION concentrations changed significantly over the course of the experiment and as a function of vertical position in the tube.

For the 6.6 nm particles, as shown in Fig. 7(a), there is a notable increase in the local SPION suspension concentration at the positions within the gap between the pole pieces (positions 3–7). At positions 4 and 5, there is a twofold increase relative to the initial value. In contrast, positions 1, 2, 8, and 9 displayed a decrease in the SPION concentration compared to the initial value. After 10.5 hours, there was a noticeable change in color as a function of position within the capillary. In particular, the suspension appeared somewhat darker between the pole pieces when compared to the regions above and below the magnet's pole pieces (Fig. S5†).

For the 13.6 nm particles, the concentration decreased to 5–20% of the initial concentration at all nine locations after a 100-minute exposure to the magnetic field. The concentration profile is similar in shape to that observed for the smallest particles, with positions 4–6 having significantly higher concentrations than at the other positions. The observed overall decrease in concentration is because the particles undergo strong horizontal motions in the direction of the magnetic flux, *i.e.*, towards the pole pieces. This led to visible particle deposition on the channel walls, as well as an obvious change in the color of the solution – from an initial dark brown/black to a final yellow – in the center of the tube (Fig. S5†). Since the X-ray beam passed through the central region of the capillary tube, it is reasonable that a substantial reduction in concentration was observed at all nine locations in the center of the channel.

**Fig. 8:**
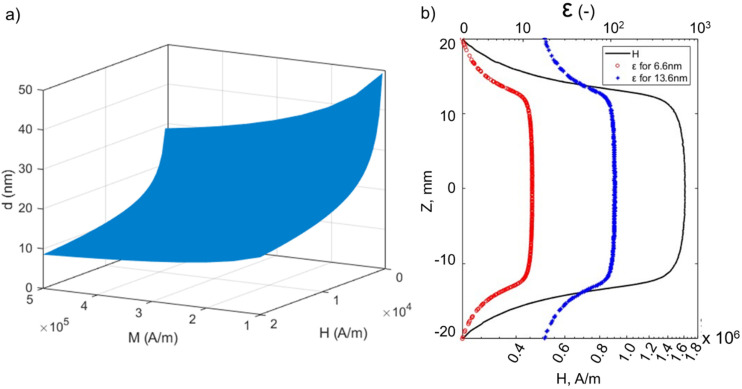
(a) Particle diameter, *d*, required to achieve *ε* = 1 (eqn (8)), at room temperature as a function of magnetic field intensity, *H*, and magnetization, *M*. (b) The values of *H* and the corresponding *ε* values for the 6.6 nm and 13.6 nm SPIONs as a function of vertical position (*z*) along the centerline of the capillary tube.

The SAXS observations suggest that even ultra-small (<10 nm) SPIONs can be magnetically manipulated in permanent magnet-based devices in a non-cooperative manner – a task that has been considered extremely challenging when these small particle sizes are exposed to the relatively small gradients imposed by conventional separators. To confirm the underlying mechanism for the separation observed in the SPION suspension, we calculated the ratio between the magnetic energy and thermal energy, denoted as *ε*, using eqn (8). Fig. 8(a) is a 3-D plot of the *ε* = 1 surface that illustrates the correlation between particle diameter (*d*), magnetization (*M*), and magnetic field intensity (*H*). The plot reveals that to successfully separate particles smaller than 10 nm, the magnetic field strength must exceed a certain threshold to surpass the disruptive effect of thermal energy, (when the conditions are above the surface as shown in Fig. 8a). Since conventional magnetic separators commonly employed for separating SPIONs typically operate at field strengths below 1 T, the task becomes exceedingly challenging.^29,50^

In our system, the magnetic field intensity distribution within the tubes is not uniform, and we focused our analysis on the particles located in the central region of the tube. The magnetic field intensity, *H*, along the tube centerline, is shown as a function of the *z*-axis position in Fig. 8(b), and the corresponding *ε* values for the 6.6 nm and 13.6 nm particles are included in Fig. 8(b). The figure illustrates that *ε* values are larger than 1 for both particle sizes for the particles positioned on the centerline of the tube along its entire length. This observation suggests that, under the experimental conditions, our system provides a sufficiently high field intensity to magnetically saturate the SPIONs, along with sufficiently high field gradients to impose a significant magnetophoretic force on them. Consequently, the forces imposed on the SPIONs surpass the thermal energy and Brownian forces, thus enabling their separation from the solvent.

### 4.3. Magnetic separation kinetics

**Fig. 9:**
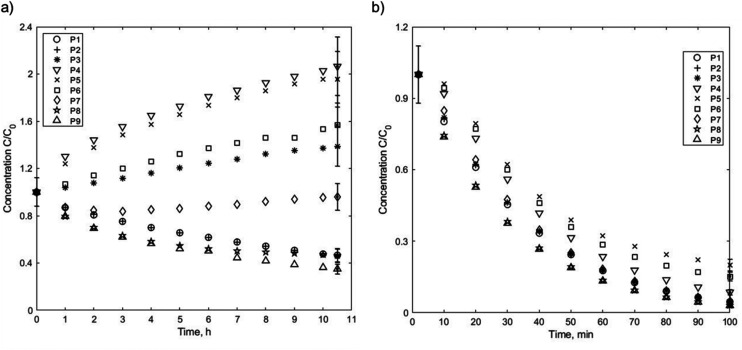
Temporal evolution of the ratio of the SPION volume fraction between the initial state and various time intervals at nine positions for particles with diameters of (a) 6.6 nm and (b) 13.6 nm. Representative error bars are shown for *t* = 0 and the end of the measurements.

Fig. 9 depicts the measured changes in the relative particle concentration as a function of time and position in the capillary tube as the particles undergo magnetic separation. The separation kinetics of the 6.6 nm (Fig. 9(a)) and 13.6 nm (Fig. 9(b)) SPION suspensions are distinctively different, and the times over which magnetic separation occurred also differ significantly. For the 6.6 nm diameter particles, X-ray measurements were taken every 30 minutes for over 10 h. However, even within the first 30 minutes, the concentration at all nine positions exhibited a discernible change. The enrichment of particles near the central positions (P3–P6) and the decrease in concentration at the outer positions, are consistent with particle migration from the regions near the edge of the pole pieces toward the highest magnetic field intensity locations. Moreover, as depicted in Fig. 6(a), there was no evidence of particle aggregation, implying that particle–particle interactions did not affect their movement.

The larger 13.6 nm particles exhibited a different behavior during the magnetic separation, as depicted in Fig. 9(b). Separation occurred quite rapidly in the first 2 minutes, with concentrations in the outer positions reducing by 20%. After 100 minutes, the concentration at all nine positions decreased relative to the initial state, with the highest remaining concentrations near position 5. This result suggests that particles move in the vertical and the horizontal directions, which is in agreement with the direction of the magnetic forces as demonstrated in more detail below.

Both sets of experimental results show that the highest concentrations were observed at position 5 throughout the duration of the study. This finding can be attributed to the fact that particles located at positions 1 and 9, which lie outside the pole pieces, experienced the strongest magnetic field gradient in the vertical direction, as can be inferred from the rapid change in *B* in these regions, as shown in Fig. 4(b). In contrast, particles located at position 5 experienced the weakest vertical magnetic field gradient; thus, a greater proportion of the particles outside the pole pieces migrated downwards (or upwards) towards the pole pieces. Again, the measured concentrations at the center of the tube decreased because particles moved in the *x*-direction, away from the tube center, due to the magnetic gradient in the horizontal direction.

### 4.4. Numerical simulation results of the SPION separation

Numerical simulations were performed to improve our understanding of the separation mechanisms and to compare the measured changes in SPION concentration within the magnetic field to those predicted by the simulations. To minimize computational costs, the simulations assumed particle diameters of 13.6 nm and a field exposure of 6 minutes with a total population of 2000 particles. The simulation began with particles uniformly suspended in the 1.49 mm inner diameter × 36 mm long tube. As discussed previously, the absence of particle agglomeration induced by dipole–dipole interactions minimizes any influence the bulk concentration of the SPIONs might have on separation.

**Fig. 10:**
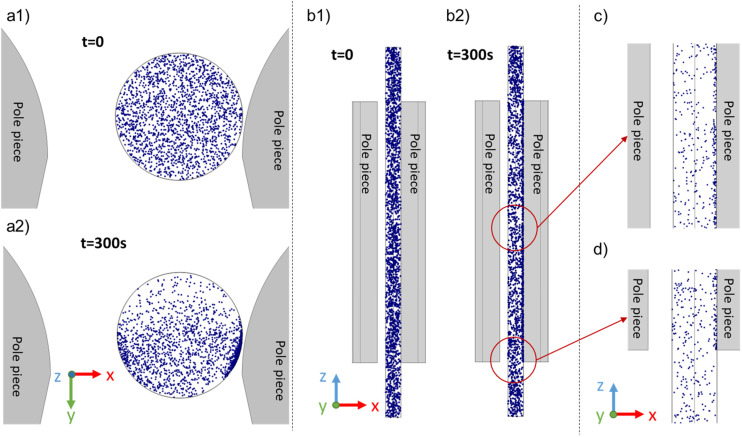
Visual representation of the 13.6 nm SPION spatial distribution obtained from the simulations, at *t* = 0 and after 300 s, respectively, in both the (a) *x*–*y* plane (top view of the tube) and (b) *x*–*z* planes. Blow-up images of particle distribution (c) between pole pieces and (d) at the bottom of the tube.

Changes in the spatial distribution of the particles throughout the simulation were captured in images, and selected images are illustrated in Fig. 10. The particle spatial distributions of all the particles in the tube in the *x*–*y* plane are shown at *t* = 0 s (Fig. 10a1) and *t* = 300 s (Fig. 10a2), respectively. Particles clearly undergo horizontal movement, accumulating and forming particle-dense regions along the walls near the pole pieces. Fig. 10b shows the particle spatial distribution in the *x*–*z* plane and the tube midpoint at the same time. Particles in the proximity of the pole pieces migrated towards the adjacent walls, while particles outside the pole pieces moved towards the edge of the pole pieces. Fig. 10(c) and (d) are enlarged images of the particle distributions after a 300 s separation between the pole pieces and at the bottom of the tube, respectively.

**Fig. 11:**
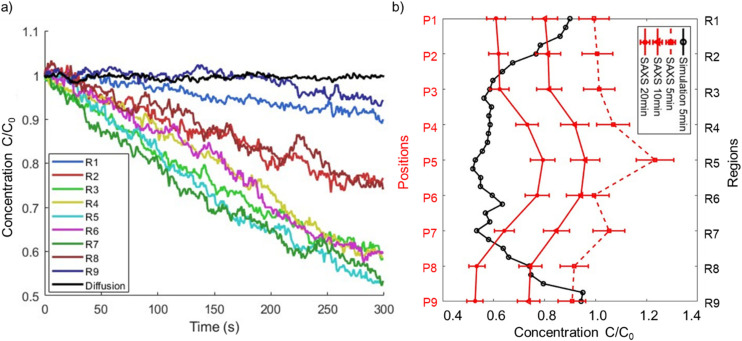
(a) Simulation results for the ratio of the number of particles as a function of time relative to the initial number of particles at *t* = 0 in different regions of the tube. The ratio does not change when particle motion is due to diffusion alone. (b) Comparative analysis of the particle count ratios: the simulation's ratio at *t* = 5 min relative to *t* = 0, and the experimental ratio at *t* = 10 min and *t* = 20 min relative to *t* = 0, as well as extrapolation for experimental ratio at *t* = 5 min. Representative error bars on *C*/*C*_0_ are shown for experimental data based on an estimated uncertainty of 6% in determining the volume fraction.

The number concentration of particles in the experimental studies (estimated as 6 × 10^15^ cm^−3^) was significantly higher than that estimated in our simulations. Thus, we used the following method to quantify the concentration changes in the vertical direction within the tube. The entire volume of the tube was partitioned into nine discrete regions. Each of these regions was discretized into volumetric segments spanning a height of 4 mm and with *x*-coordinates spanning from −0.2 mm to 1 mm. The *x*-range was deliberately chosen to be slightly less than the radius of the tube to better mimic the narrow (∼0.1 mm) X-ray beam. As an illustrative example, the first (top) region was designated “R1”, corresponding to the volumetric segment with *x*-coordinates ranging from −0.2 mm to 1 mm, *y*-coordinates ranging from 10.05 mm to 11.54 mm, and *z*-coordinates ranging from 14 mm to 18 mm. The number of particles within each region was then tracked with time. The results are depicted in Fig. 11(a), as the ratio of the number of particles present in each region at various time points to the initial count at *t* = 0. To demonstrate that magnetophoresis played an important role in the separation process, we also simulated particle motion considering only diffusion, and included these results as the solid black line in Fig. 11. After ∼50 s, the curves that consider magnetophoresis deviated from the pure diffusion curve, and furthermore the former depend on the location within the tube and, thus, on the local magnetic conditions. This provides clear evidence that the observed separation and distinct particle behavior across different regions are primarily attributed to magnetophoresis rather than Brownian diffusion or gravity. Neglecting particles close to the wall, leads to a 20–40% decrease in the total number of particles in the magnetophoretic calculations in relation to the pure diffusional calculation.

Furthermore, Fig. 11(b) compares the simulation results at *t* = 5 min to the experimental data acquired at *t* = 10 min and *t* = 20 min (corresponding to the initial measurement performed using SAXS). To better compare simulation and experiments, we interpolated the measured results to estimate the expected concentration at *t* = 5 min. These values correspond to the dotted line in Fig. 11(b). In contrast to the experimental results, at *t* = 5 min, the simulations predict substantially lower concentrations within the inter-pole piece regions but comparable values in the regions situated outside the pole pieces. Alternatively, extrapolating the trends in Fig. 11(a) to *t* = 10 min would lead to lower concentrations in R1 and R9, which would be in better agreement with the experimental values. In the inner regions (R4–R6), the difference in the simulation and experimental time cannot explain the observed differences. Rather, they suggest that the model overpredicts the horizontal motion of the particles relative to their vertical motion. If particles had a lower horizontal velocity relative to the vertical velocity, the loss of particles to the wall in the central regions of the tube would be slowed down without affecting the removal of particles from the outer regions, and particles could “build up” in the inner regions relative to the outer regions. Given that the model is based on a somewhat idealized pole geometry and tube location, this discrepancy may reflect inaccuracies in the calculated horizontal magnetic field gradients relative to the experimental values.

**Fig. 12:**
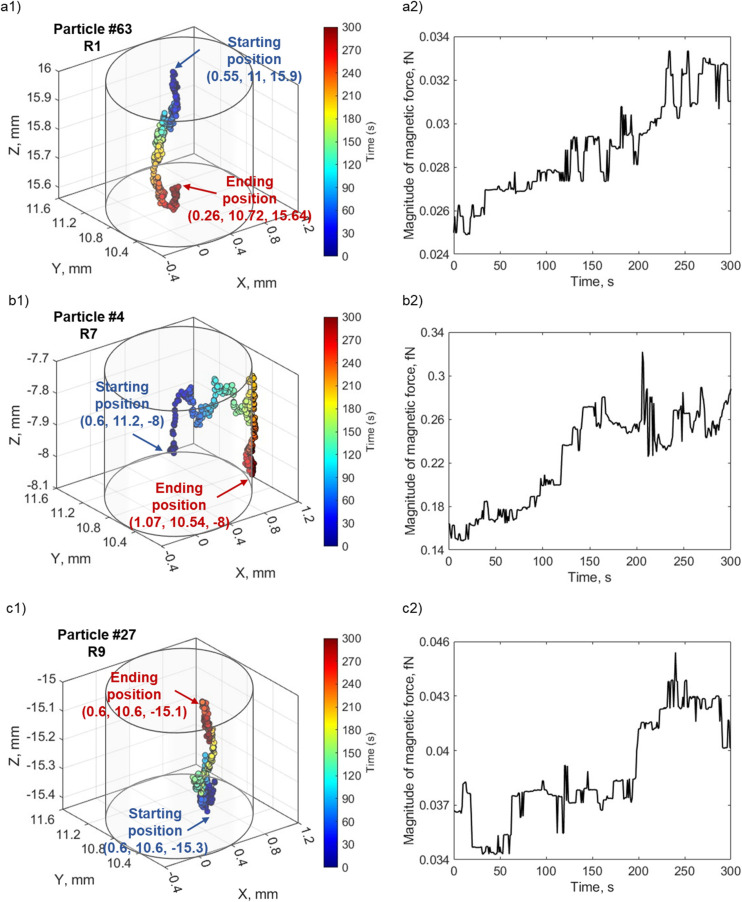
3-D trajectories of individual particles located in (a1) R1, (b1) R7, and (c1) R9 at *t* = 0. The colored dots trace the particle trajectory at sequential time intervals. The initial and final positions are indicated. The magnitude of the magnetic forces acting on the respective particles at successive temporal steps, are shown in (a2), (b2) and (c2).

To visualize the dynamic motion of particles influenced by magnetophoresis, Fig. 12(a1)–(c1) illustrate the 3-D trajectories for three different particles. The spatial coordinates of individual particles are graphed at discrete time points indicated by the change in color. The starting and ending points of each particle's trajectory are also indicated, and the magnitude of the net magnetophoretic forces exerted on these particles is shown as a function of time in Fig. 12(a2)–(c2). Particle #63 started in the top region of the tube (R1), outside the pole pieces, and moved down toward the pole pieces under the influence of the magnetic field gradient. The average magnetophoretic force acting upon this particle is 0.03 fN, and the force acting on it increases as it moves closer to the poles. The magnetophoretic force is roughly three orders of magnitude larger than the gravitational force of 5.34 × 10^−5^ fN. Conversely, particle #44, Fig. 12(b1) and 12(b2), initially positioned in between the pole pieces (R7), undergoes both upward and downward motion as it migrates toward the wall adjacent to the pole piece, followed by downward motion along the inner surface of the capillary. The magnetic force experienced by particle #44 increases as it approaches the pole piece and peaks at 0.3 fN. These forces are an order of magnitude higher than those experienced by particle #63 and can be attributed to particle #44's exposure to the elevated magnetic field intensity and field gradient. Finally, particle #27, Fig. 12(c1) and 12(c2), originates at the low end of the tube beyond the pole piece (R9). The upward movement of this particle reflects the fact that the magnetophoretic force is approximately 2 orders of magnitude larger than the gravitational force. Thus, the particle can easily overcome gravitational forces. In all of the cases, thermal forces randomize the path taken but not the overall particle motion.

**Fig. 13:**
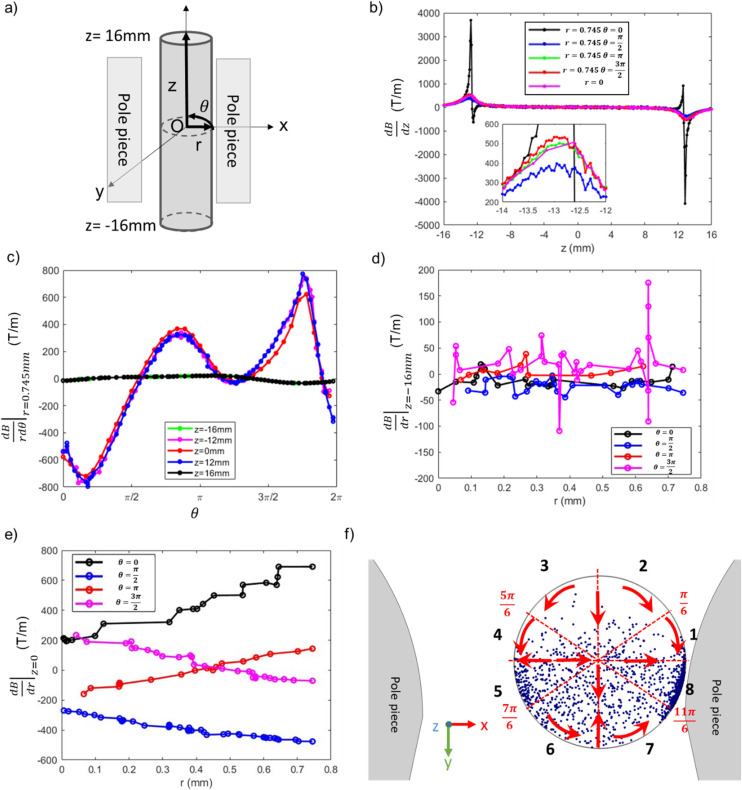
Magnetic field gradient in cylindrical system. (a) Cylindrical coordinates converted from the global Cartesian coordinates. (b) 
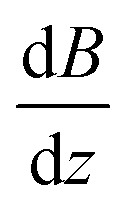
 with respect to the axial coordinate “*z*” at *r* = 0 (the central region of the tube) and at *r* = 0.745 mm (adjacent to the tube's inner wall). (c) 
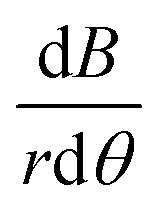
 as a function of *θ* at a radial distance of *r* = 0.745 mm (at the inner wall of the tube). (d) 
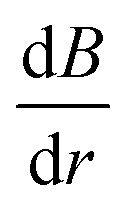
 as a function of *r*, at *z* = −16 mm. (e) 
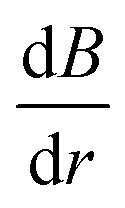
 as a function of *r*, at *z* = 0 mm. (f) Visual representation of the 13.6 nm SPION spatial distribution obtained from the simulations after 300 s within the pole pieces in *x*–*y* planes, divided in 8 regions.

To better understand the range of magnetophoretic behavior of observed for particles in different regions of the tube, the spatial distribution of magnetic field gradients within the tube is required. A transformation from the global Cartesian coordinate system was implemented to generate the cylindrical coordinates depicted in Fig. 13(a). The present cylindrical coordinate system is characterized by the parameters *r*, *θ*, and *z*, with its origin established at the coordinates *x*, *y*, *z* = (0.4, 10.795, 0), as derived from the pre-existing coordinate system. To understand the correlation between magnetic gradient and its impact on particle trajectory, we focus on two distinct spatial domains: the external regions outside the pole pieces (|*z*| > 12 mm) and the internal regions within the pole pieces (|*z*| ≤ 12 mm).

The vertical magnetic field gradient above or below the pole pieces (|*z*| > 12 mm), 
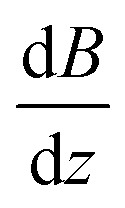
 (Fig. 13(b)) is much more important than the gradients in the angular (Fig. 13c) or radial (Fig. 13d) directions. The values of 
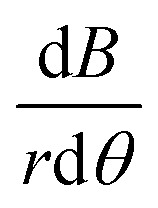
 along the inner wall of the tube (*r* = 0.745 mm) are always between −30 and 30 T m^−1^, whereas the values of 
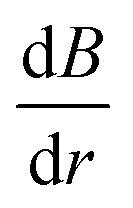
 always lie between −50 to 50 T m^−1^. The strongest vertical magnetic field gradients are close to *z* = ±13 mm, *i.e.*, in the regions near the pole pieces’ top and bottom edges. At these axial positions, 
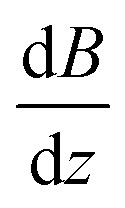
 is on the order of 300–500 T m^−1^ for most values of *r* and *θ*. The striking exception is near the inner wall of the tube when *θ* = 0, *i.e.*, in the region closest to the pole pieces. Here 
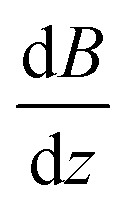
 is predicted to reach up to 3800 T m^−1^. These observations support our hypothesis that particles situated outside the pole pieces encounter significantly higher vertical magnetic forces than in-plane magnetic forces. Consequently, the particles largely move in a vertical direction, a phenomenon graphically illustrated in Fig. 12(a1) and (c1). The horizontal perturbations shown in the motion of these two particles can be largely attributed to Brownian motion.

While particles outside the pole pieces experience mostly vertical magnetic field gradients, particles within the pole pieces are primarily subjected to forces acting in the radial and angular directions. Between the pole pieces (Fig. 13(b), |*z*| < 12 mm), the magnitude of the vertical field gradient is always less than 100 T m^−1^. Magnetic forces acting in the angular direction are proportional to 
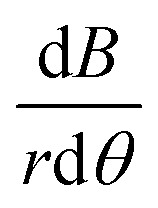
, and as shown in Fig. 13(c), the changes in 
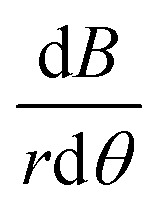
 with *θ* are not a function of *z* along the inner wall of the tube (*r* = 0.745 mm). The complex shape of the 
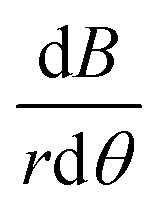
 curve in the region between the pole pieces is attributed to the asymmetric shape of the pole pieces and the position of the tube with respect to the pole pieces. Some differences in the curves may stem from the mesh limitations employed in our simulation and the coordinate transformation from Cartesian to cylindrical coordinates. However, all three curves, exhibit extrema at 
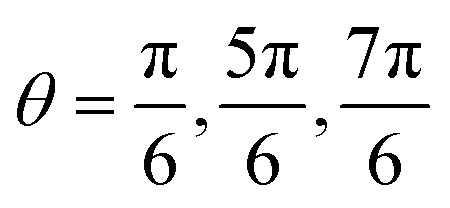
 and 
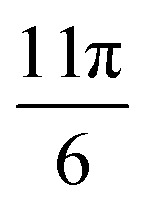
. Finally, Fig. 13(e) illustrates the changes in 
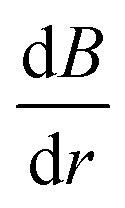
 as a function of *r* for *z* = 0 and selected values of *θ*.

To understand how the interplay between 
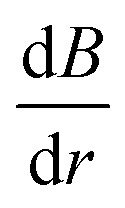
 and 
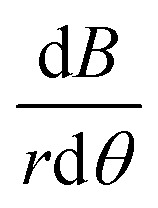
 can lead to the experimentally observed and modeled deposition patterns, Fig. 13(f) shows the particle spatial distributions in the *x*–*y* plane integrated over all particles residing between the poles of the magnet (|*z*| < 12 mm) at *t* = 300 s (Fig. 10a2). The region is segmented into eight distinct sectors, with sectors defined by the locations of the extrema as well as the angles corresponding to the *x* and *y* axes. The red arrows indicate the direction of the forces acting on the particles due to 
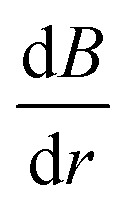
 and 
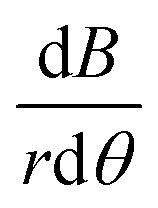
. For particles situated on the inner surface of the tube within regions 1–4 (0 < *θ* < π), the angular magnetic gradient acts to move the particles toward the closest pole piece located at *θ* = 0 and *θ* = π. Consequently, these particles aggregate in regions 5 and 8. Simultaneously, the radial magnetic gradient generally moves the particles towards the center of the tube. Similarly, particles positioned along the inner wall in regions 6 and 7 want to migrate towards the nearest pole piece, ultimately accumulating in region 8. This analysis explains how particles move to produce the higher particle densities observed in regions 5 and 8, *i.e.*, the regions closest to the pole pieces. These density variations could not be discerned in X-ray measurements that were conducted at the midsection of the tube. However, at the end of the experiments involving the 13.6 nm and 30 nm particles, it was clear that particles preferentially collected on the sides of the tube closest to the pole pieces, leaving relatively clean surfaces along the beam path. Furthermore, the radial and angular gradient analyses explain the lower particle density observed in regions 2 and 3 relative to regions 5 and 6.

## 5. Conclusions

The unique characteristics of SPIONs render them suitable for a wide range of applications. However, the efficient separation of SPIONs smaller than 10 nm from solutions faces significant challenges due to the influence of thermal energy. Conventional magnetic separators based on permanent magnets typically provide magnetic intensities of up to 1 T and gradients below 100 T m^−1^, which are insufficient for the SPIONs in this size range to overcome thermal energy. Consequently, more robust magnetic separators with greater power are necessary. In our previous investigation, we successfully achieved the separation of SPIONs ranging from 5 nm to 30 nm utilizing high gradient, permanent magnet-based, and matrix-free separators. We hypothesized that the particles experienced dipole–dipole interactions, leading to the formation of agglomerates that subsequently sediment inside the device.

To gain deeper insights into the underlying mechanism governing the separation, in this work we designed a magnetic device wherein SPIONs were positioned between magnetic pole pieces. SAXS was used to measure both particle size distribution parameters and the relative concentration of the suspensions. SPIONs with average sizes of 6.6 nm, 13.6 nm, and 27.5 nm all exhibited separation by migrating towards the magnet pole pieces, following the magnetic flux lines. Nonetheless, SAXS measurements showed that the 6.6 nm and 13.6 nm SPIONs did not form aggregates during the process. Consequently, separation can be attributed to non-cooperative magnetophoresis; the presence of a high magnetic field and magnetic field gradient provide adequate magnetic force to overcome Brownian forces. In contrast, the 27.5 nm SPIONs located between the magnet pole pieces organized into superstructures due to magnetic dipole–dipole interactions. The latter did not, however, precipitate to the bottom of the tube. Our analysis confirms that it is feasible to separate particles with dimensions below 10 nm using matrix-free, permanent magnet-based devices when the magnetic field strength and magnetic field gradient are sufficient to achieve magnetic forces comparable or greater than those due to thermal energy.

This work also demonstrates that SAXS is a potent tool for exploring the aggregation status of the nanoparticle suspensions both before and during separation and for unraveling the separation mechanisms of small SPIONs under 30 nm. In particular, SAXS measurements can ensure that initial nanoparticle solutions are not aggregated prior to any subsequent treatment. We note that the choice of solvent also influences the stability of the SPIONs in suspension. Notably, chloroform has the potential to induce pre-aggregation of particles, detrimental to several processes in biomedical applications and similar fields.

Finally, numerical simulations successfully simulated the magnetic field generated inside our separator and they were able to model the magnetophoretic behavior of nanometer-sized particles, although not all of the predictions matched the experimental results. We conclude that the use of such computational models will enhance our ability to design magnetic separators tailored to specific requirements and predict the separation behavior of particles with varying properties.

## Conflicts of interest

There are no conflicts to declare.

## Supplementary Material

NR-016-D3NR05589B-s001

NR-016-D3NR05589B-s002

NR-016-D3NR05589B-s003
