# Supplementary material for: Numerical modeling and in situ small angle X-ray scattering characterization of ultra-small SPION magnetophoresis in a high field and gradient separator
Source: Nanoscale. 2024 Mar 6;16(14):7041–57. doi: 10.1039/d3nr05589b (PMC10993306; doi:10.1039/d3nr05589b)
Supplement: NR-016-D3NR05589B-s001 [file NR-016-D3NR05589B-s001.pdf]

Electronic Supplementary Material (ESI) for Nanoscale.
This journal is © The Royal Society of Chemistry 2024

Supplementary information

Numerical modeling and *in-situ* Small Angle X-ray Scattering characterization of ultra-small SPION magnetophoresis in a high field and gradient separator

Xian Wu,[a] Hyeon Choe,[a] Jacob Strayer,[a] Jenifer Gómez-Pastora,[b] Maciej Zborowski,[c] Barbara Wyslouzil,[a,d] Jeffrey Chalmers[a,*]

[a]William G. Lowrie Department of Chemical and Biomolecular Engineering

The Ohio State University

151 West Woodruff Avenue, Columbus, OH 43210

[b]Department of Chemical Engineering

Texas Tech University

2500 Broadway Lubbock, Texas 79409

[c]Department of Biomedical Engineering

Cleveland Clinic

9500 Euclid Avenue, Cleveland, OH 44195

[d]Department of Chemistry and Biochemistry

The Ohio State University

100 West 18th Avenue, Columbus, OH 43210

Table of contents

Figure S1. CAD drawing of the magnet separator.

Figure S2. Final SAXS scattering spectra measured for particle suspensions after exposure to the magnetic field for the time indicated. Model fits assume scattering from a polydisperse (Gaussian) collection of spheres. a) 6.6 nm (t = 10.5 h), b) 13.6 nm (100 min) and c) 27.5 nm (30 min).

Figure S3. X-ray scattering spectra for 6.6 nm SPIONs at the beginning of the experiment and after 10.5h exposure to the magnetic field.

Figure S4. X-ray scattering spectra for 13.6 nm SPIONs at the beginning of the experiment and after 100min exposure to the magnetic field.

Figure S5: The changes in the color of the SPION suspensions reflect the observed changes in C determined from SAXS measurements. a) and c) are for the 6.6 nm particles, b) and d) are for the 13.6 nm particles.

Video 1. Animation of particle trajectories in x-y plane obtained from Comsol

Video 2. Animation of particle trajectories in x-z plane obtained from Comsol

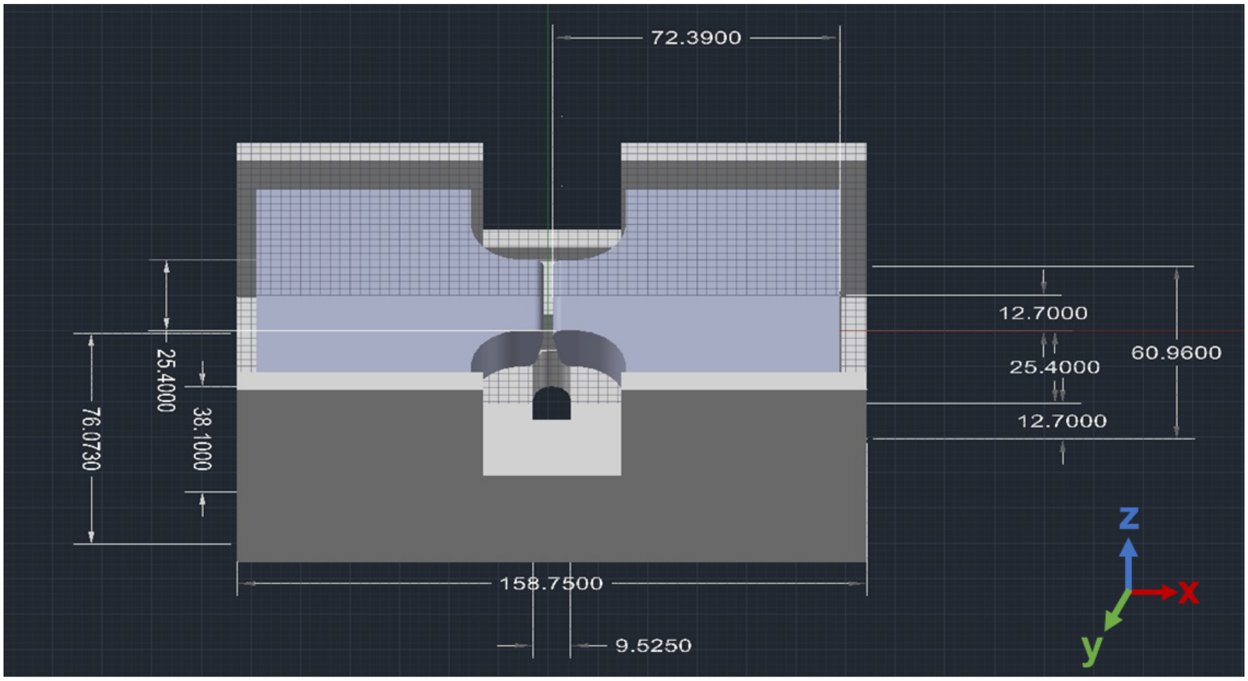

Figure S1. CAD drawing of the magnet separator.

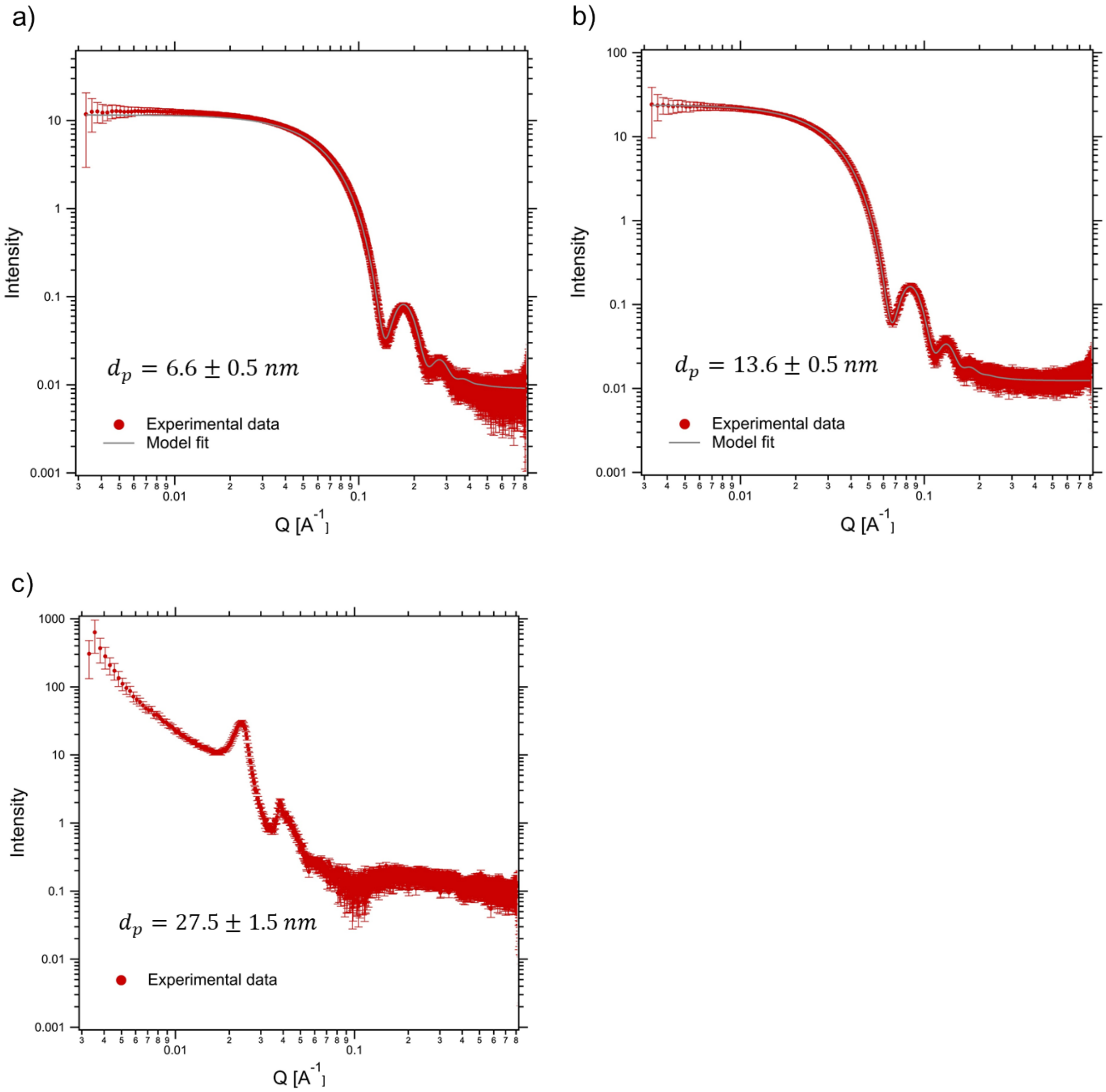

Figure S2. Final SAXS scattering spectra measured for particle suspensions after exposure to the magnetic field for the times indicated. a) 6.6 nm (t = 10.5 h), b) 13.6 nm (100 min) and c) 27.5 nm (30 min). For the two smaller particle sizes, the model fits assume scattering from a polydisperse (Gaussian) collection of uniformly dispersed spheres. For the largest particle size, the reported size is based on fitting the scattering data collected prior to placing the sample in the magnetic field.

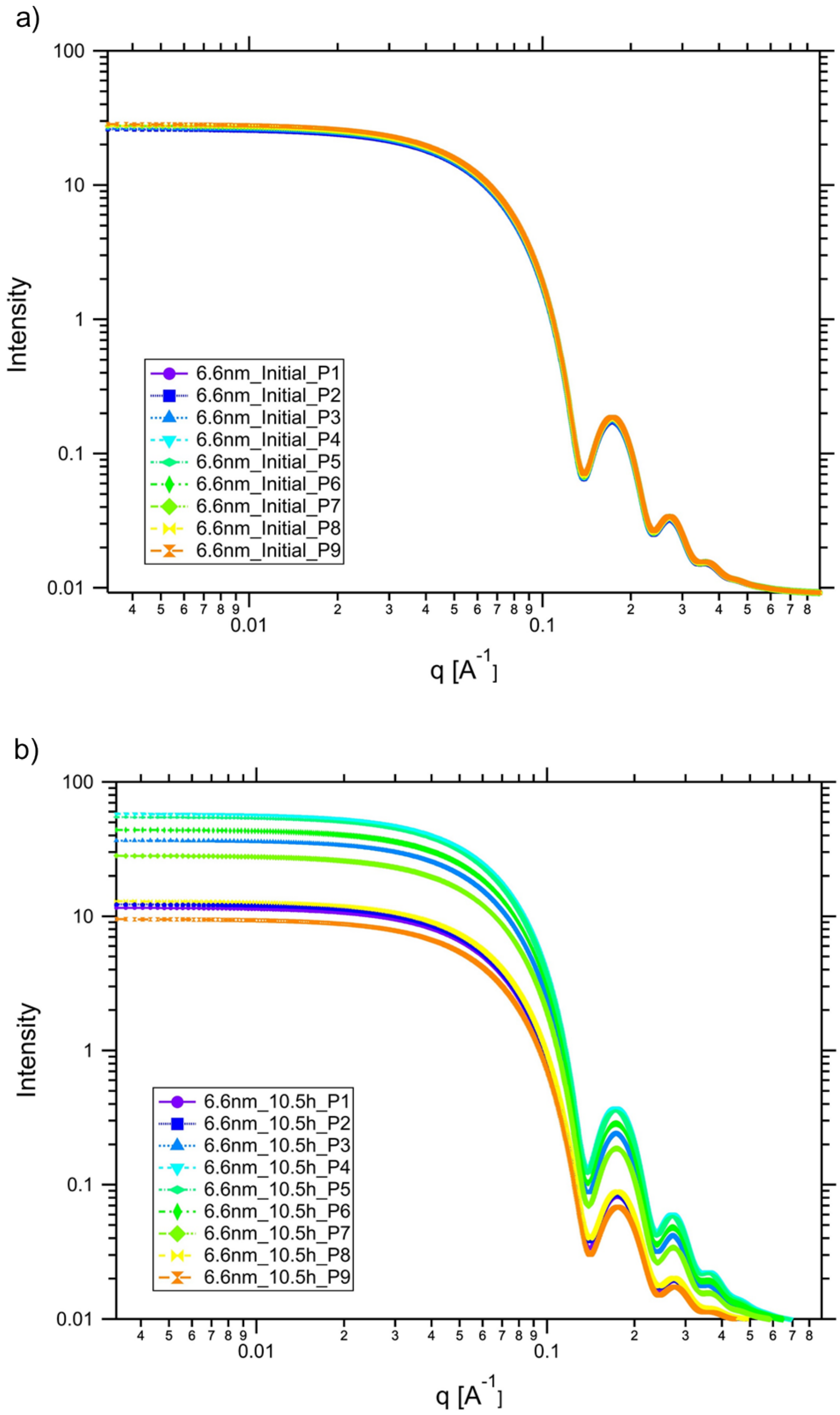

Figure S3. X-ray scattering spectra for 6.6 nm SPIONs at a) the beginning of the experiment and b) after 10.5h exposure to the magnetic field.

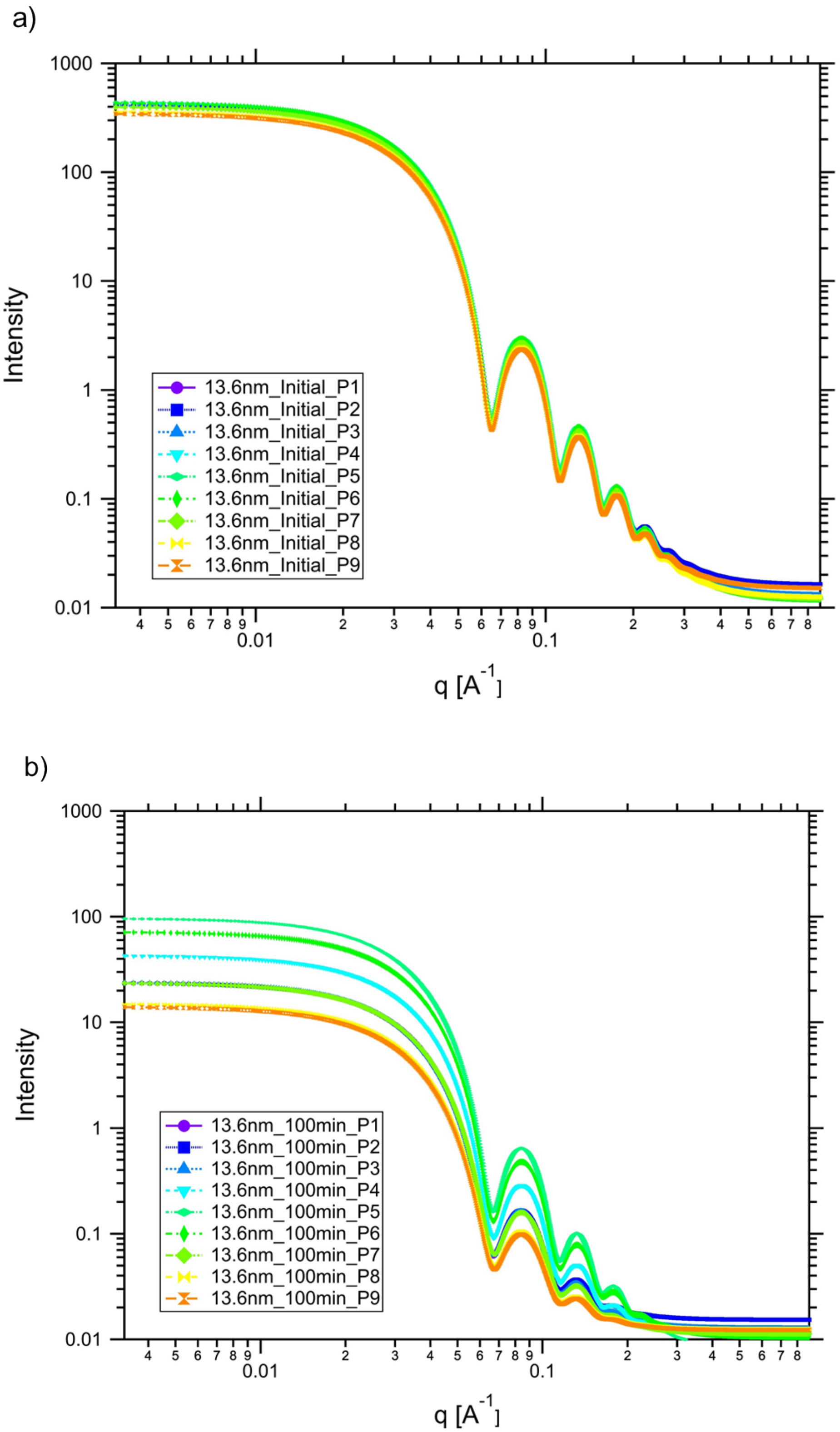

Figure S4. X-ray scattering spectra for 13.6 nm SPIONs at a) the beginning of the experiment and b) after 100min exposure to the magnetic field.

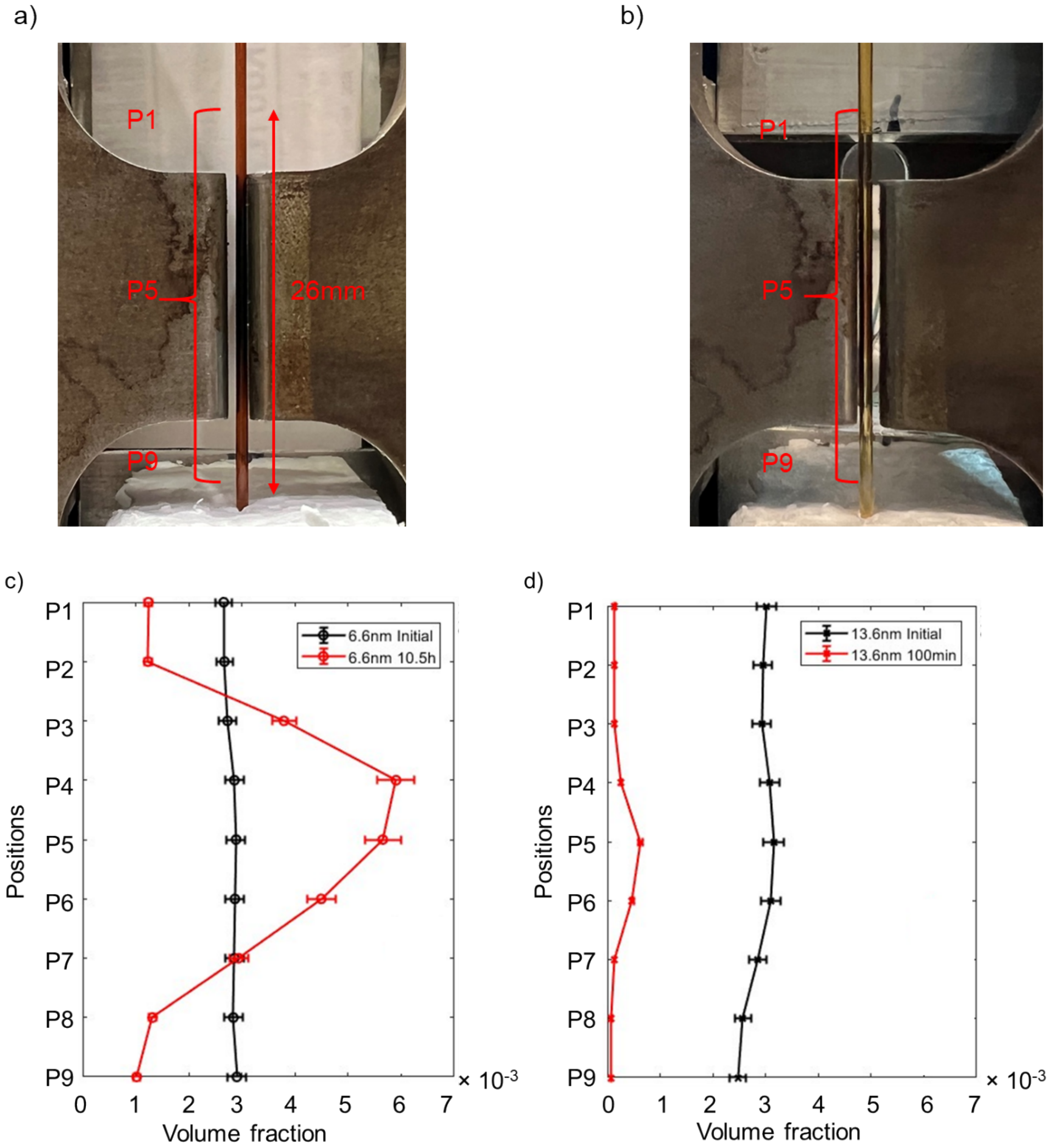

Figure S5: The changes in the color of the SPION suspensions reflect the observed changes in volume fraction determined from SAXS measurements. a) and c) are for the 6.6 nm particles, b) and d) are for the 13.6 nm particles.
